# A *myo*-inositol dehydrogenase involved in aminocyclitol biosynthesis of hygromycin A

**DOI:** 10.3762/bjoc.20.51

**Published:** 2024-03-14

**Authors:** Michael O Akintubosun, Melanie A Higgins

**Affiliations:** 1 Department of Biological Sciences, The University of Alabama, 3314 Science and Engineering Complex, Tuscaloosa, AL 35487, USA

**Keywords:** aminocyclitol, biosynthesis, hygromycin A, inositol dehydrogenase, *myo*-inositol

## Abstract

Hygromycin A is a broad-spectrum antibiotic that contains a furanose, cinnamic acid, and aminocyclitol moieties. The biosynthesis of the aminocyclitol has been proposed to proceed through six enzymatic steps from glucose 6-phosphate through *myo*-inositol to the final methylenedioxy-containing aminocyclitol. Although there is some in vivo evidence for this proposed pathway, biochemical support for the individual enzyme activities is lacking. In this study, we verify the activity for one enzyme in this pathway. We show that Hyg17 is a *myo*-inositol dehydrogenase that has a unique substrate scope when compared to other *myo*-inositol dehydrogenases. Furthermore, we analyze sequences from the protein family containing Hyg17 and discuss genome mining strategies that target this protein family to identify biosynthetic clusters for natural product discovery.

## Introduction

**Figure 1:**
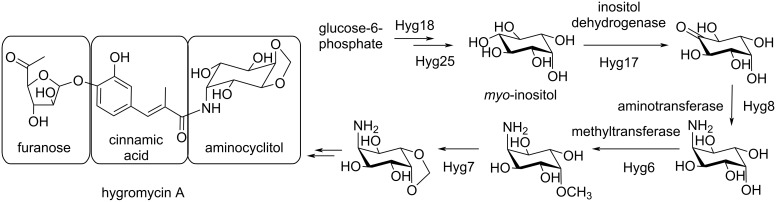
Proposed biosynthetic pathway for the aminocyclitol from hygromycin A.

Hygromycin A is a natural product that was discovered in the 1950s and is produced by the soil bacterium *Streptomyces hygroscopicus* [1]. It has broad spectrum antibiotic activity, antitreponemal activity against the pathogen that causes swine dysentery, and selective activity against the spirochete that causes Lyme disease [1–3]. It binds the large 50S ribosomal subunit to inhibit ribosomal peptidyl transferase activity [4–5] and contains three distinctive functional groups: furanose, cinnamic acid, and aminocyclitol (Figure 1). The cinnamic acid and aminocyclitol moieties directly restrict access of the amino-tRNA to inhibit peptidyl transferase activity while the furanose group does not appear to be important for target inhibition [6–7]. In addition, hygromycin A contains a unique methylenedioxy group found on the aminocyclitol that is not required for ribosome interaction and in vitro inhibition [8]. Instead, it is essential for in vivo antimicrobial activity suggesting a distinct biological function independent of ribosome binding.

The hygromycin A biosynthetic gene cluster has been identified and the biosynthesis of the aminocyclitol has been proposed (Figure 1) [8–9]. Starting from glucose-6-phosphate, the pathway is hypothesized to proceed through six steps to the final methylenedioxy-containing aminocyclitol. First, glucose-6-phosphate is thought to be converted to *myo*-inositol by the *myo-*inositol-1-phosphate synthase Hyg18 and phosphatase Hyg25. *Myo*-inositol is then proposed to be oxidized by the dehydrogenase Hyg17 to form *neo*-inosose, followed by a transamination to *neo*-inosamine-2 by the aminotransferase Hyg8. The methyltransferase Hyg6 would then install a methyl group which would set up cyclization of the methylenedioxy group by Hyg7. This biosynthetic pathway has been proposed based on gene annotations and in vivo studies [8]. However, validation by in vitro approaches or biochemical analysis of the individual enzymes is lacking. Here, we verify that Hyg17 is a *myo*-inositol dehydrogenase and show that it has a distinct substrate scope. In addition, we use sequence similarity networks to compare Hyg17 sequences with other members of the oxidoreductase family and inositol dehydrogenases and discuss specialized genome mining approaches using these sequences to identify new natural product biosynthetic clusters.

## Results and Discussion

### Hyg17 enzyme activity

**Figure 2:**
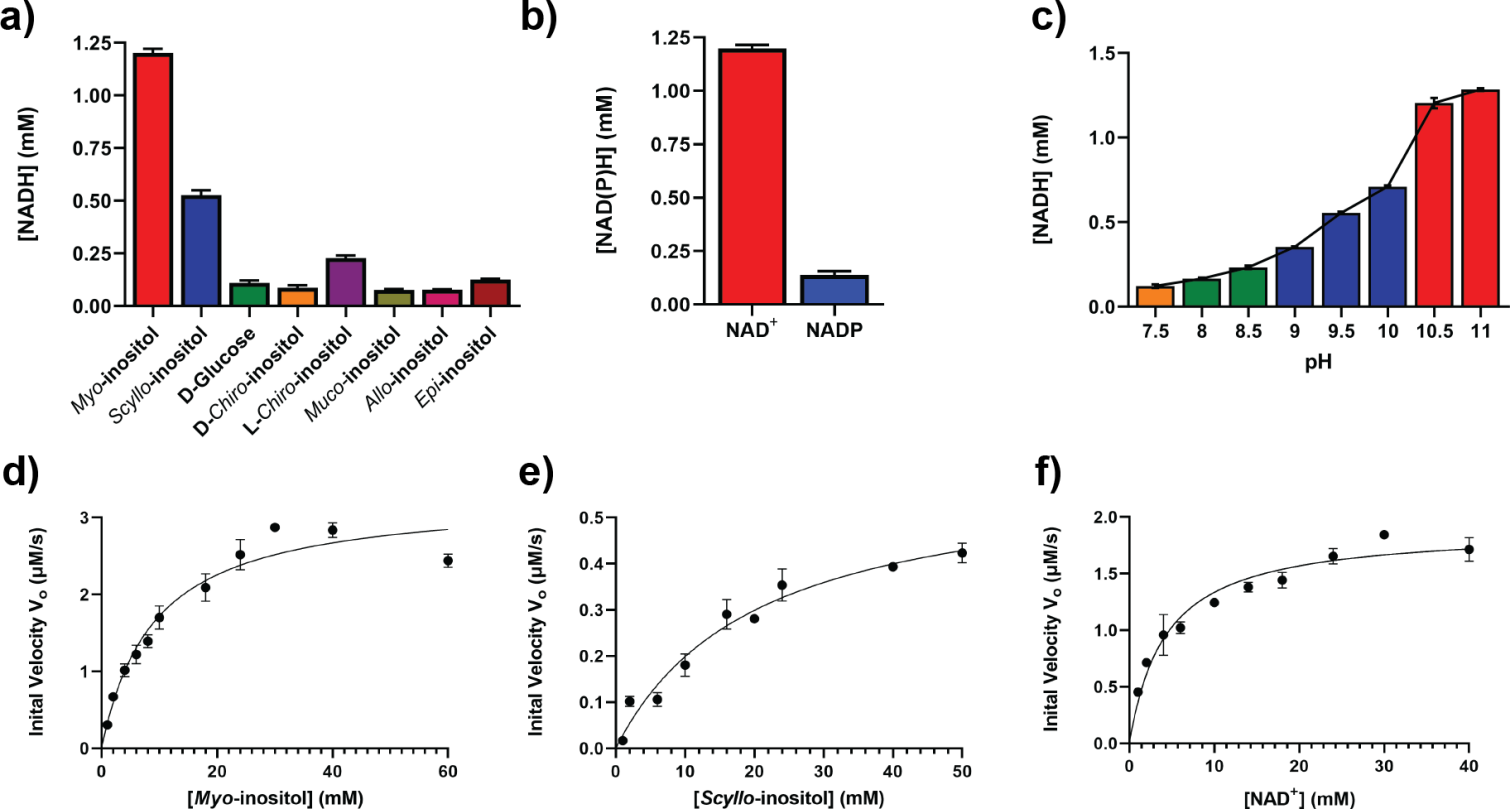
Hyg17 activity. Reactions with Hyg17 and (a) various inositols with NAD^+^, (b) *myo*-inositol with NAD^+^ or NADP, and (c) *myo*-inositol at different pH values (orange is HEPEs, green is Tris, blue is CHES, and red is CAPS). NAD(P)H concentrations were measured after 20 minutes. Michaelis–Menten plots for Hyg17 using varying concentrations of (d) *myo*-inositol, (e) *scyllo*-inositol, and (f) NAD^+^. Reactions were monitored for NADH.

We found that Hyg17 formed inclusion bodies when recombinantly produced by various *E. coli* expression strains. However, we were able to obtain pure soluble protein when expressing Hyg17 in a *Rhodococcus* expression system (Supporting Information File 1, Figure S1) [10–11]. According to the proposed hygromycin A biosynthetic pathway, Hyg17 is a *myo*-inositol dehydrogenase. These types of enzymes typically use NAD^+^ as a cofactor [12–13]. So, we first tested Hyg17 with *myo*-inositol and NAD^+^ and found that it was able to produce NADH, suggesting it can function as a *myo*-inositol dehydrogenase (Figure 2a). Since this assay tests for the formation of NADH, we are assuming the formation of a ketone product. However, further experiments are required to verify this assumption and determine which inositol position is being oxidized. When we tested Hyg17 with *myo*-inositol and NADP, we found no activity, showing that Hyg17 is NAD^+^-dependent (Figure 2b). Although this is consistent with native *myo*- and *scyllo*-dehydrogenases LcIDH1 and LcIDH2 from *Lactobacillus casei* [12], there have been reports of a genetically engineered NAD-specific dehydrogenase that has been converted to an efficient NADP-dependent enzyme [14]. Next, we determined the optimal pH of Hyg17. We observed increased activity as the pH increased, with the greatest activity observed at pH 10.5–11 (Figure 2c and Supporting Information File 1, Figure S2). Similarly, LcIDH1 and LcIDH2 have an optimum pH of 9.3 and 9.5, respectively, while the *myo*-inositol dehydrogenase from *Bacillus subtilis*, BsIDH, has an optimal pH between 9.5–10 [12–13]. We also compared product formation between reactions with Hyg17 or BsIDH and *myo*-inositol using thin-layer chromatography (Supporting Information File 1, Figure S3). We found that both enzymes generated a ketone product with identical retention factors. Further studies are required to determine if Hyg17 oxidizes the same position of *myo*-inositol as BsIDH and other *myo*-inositol dehydrogenases.

We then tested the substrate scope for Hyg17. Activity was best using *myo*-inositol as substrate further validating that Hyg17 is a *myo*-inositol dehydrogenase (Figure 2a). We also found there was reduced activity with *scyllo*-inositol, minimal activity with ʟ-*chiro*-inositol and no activity with ᴅ-glucose, ᴅ-*chiro*-inositol, *epi*-inositol, *muco*-inositol, and *allo*-inositol. By comparison, other *myo-*inositol dehydrogenases typically do not have activity on *scyllo*-inositol but can have reduced activity on ᴅ-*chiro*-inositol, ᴅ-glucose, ᴅ-xylose and 4-*O*-benzyl-*myo*-inositol [12–13,15–17]. However, *scyllo*-inositol dehydrogenases are active on *scyllo*-inositol and *myo*-inositol to a lesser extent [16,18]. Altogether, this suggests that Hyg17 can accommodate different substrates when compared to known *myo*-inositol dehydrogenases and has a more similar substrate scope to *scyllo*-inositol dehydrogenases.

We performed kinetics analysis for Hyg17 with *myo*- and *scyllo*-inositol (Table 1 and Figure 2d,e). The *K*_M_ value for Hyg17 with *myo*-inositol was 9.0 ± 1.1 mM, which is relatively high. However, this is similar to other *myo*-inositol dehydrogenases whose *K*_M_ values are also in the mM range [12,16]. Hyg17 showed a higher catalytic efficiency of 366.7 ± 46.96 M^−1^ s^−1^ with *myo*-inositol as compared to the 29.6 ± 5.28 M^−1^ s^−1^ for *scyllo*-inositol. This reduced catalytic efficiency can be attributed to the reduced *k*_cat_ and increased *K*_M_ for *scyllo*-inositol over *myo*-inositol. We also found that the catalytic efficiency for NAD^+^ was 452.4 ± 54.49 M^−1^ s^−1^ (Table 1 and Figure 2f).

**Table 1:** Kinetic parameters for Hyg17.

Substrate/cofactor	*k*_cat_, s^−1^	*K*_M_, mM	*k*_cat_/*K*_M_, M^−1^ s^−1^

*myo*-inositol	3.3 ± 0.13	9.0 ± 1.1	366.7 ± 46.96
*scyllo*-inositol	0.60 ± 0.044	20.3 ± 3.3	29.6 ± 5.28
NAD^+^	1.9 ± 0.057	4.2 ± 0.49	452.4 ± 54.49

### Sequence similarity network

**Figure 3:**
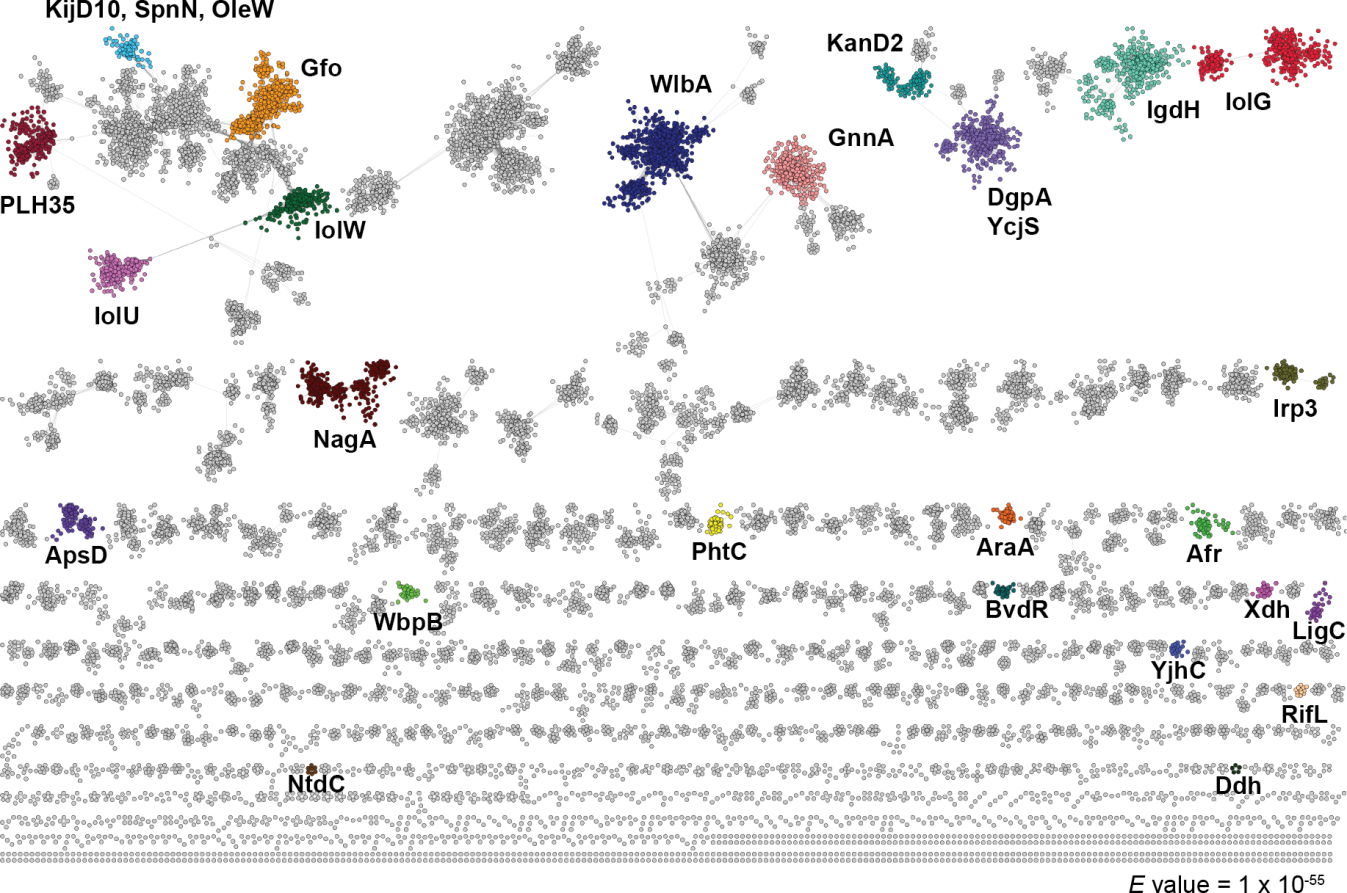
SSN for PF01408. Clusters with characterized enzymes are shown in different colors and labeled with the protein name. Functional details for characterized enzymes in PF01408 can be found in Supporting Information File 1, Table S1.

We generated a sequence similarity network (SSN) for the protein family PF01408, in which Hyg17 is a member (Figure 3). PF01408 is classified as an oxidoreductase with NAD-binding Rossmann fold family and contains over 340,000 sequences. Many of the family members act as sugar dehydrogenases with diverse sugar substrates (Supporting Information File 1, Table S1). These enzymes can have distinct biological functions, such as sugar metabolism and LPS biosynthesis [19–23]. By contrast, other members are involved in natural product biosynthetic pathways similar to Hyg17 [24–26]. Although many of the PF01408 enzymes have reported activities, the SSN shows there are still several clusters whose activity remain unknown, suggesting potential for new enzyme discovery within this family of enzymes.

**Figure 4:**
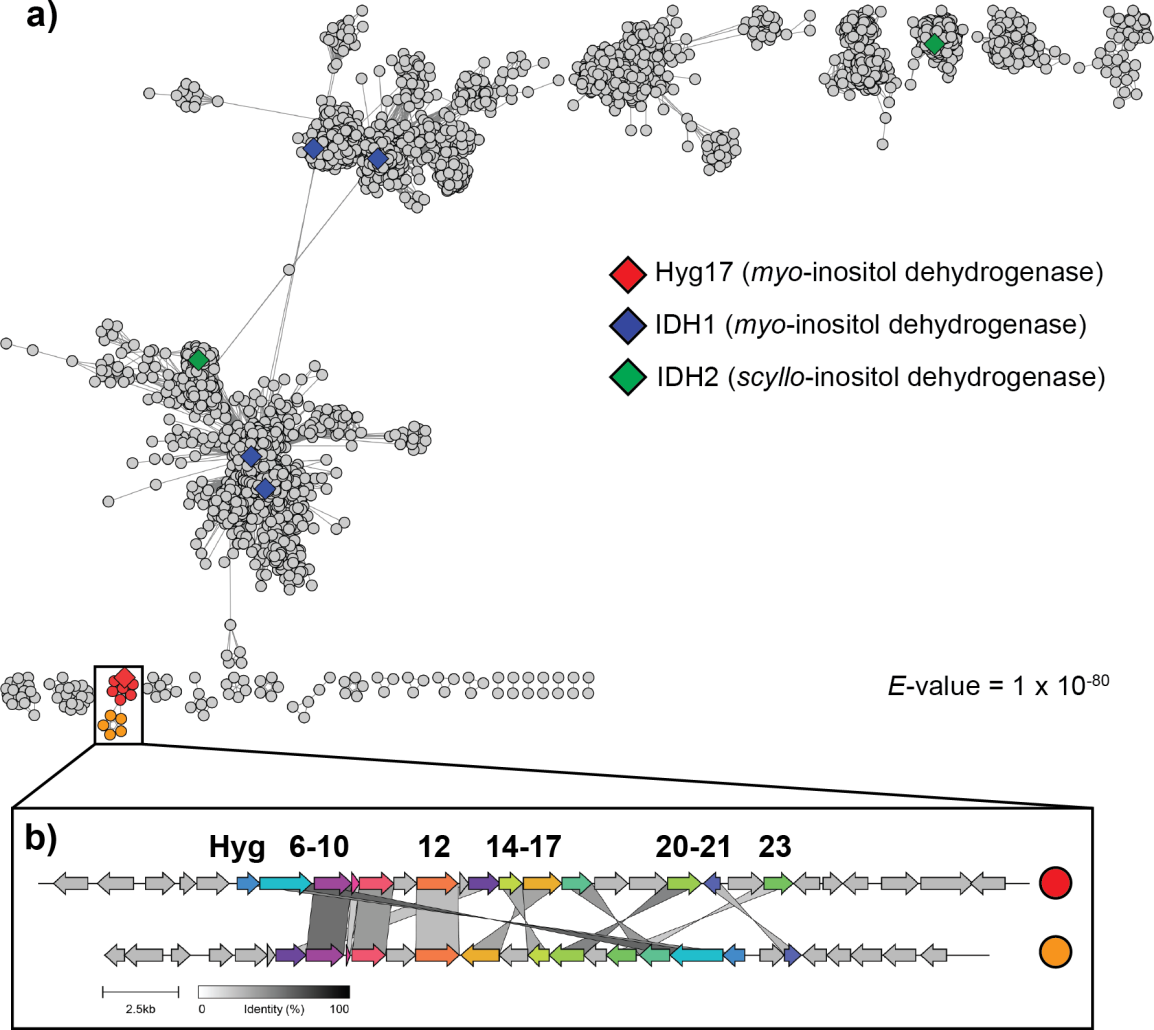
(a) SSN for inositol dehydrogenases. (b) Comparison of the hygromycin A (red) and hygromycin A-like (orange) biosynthetic gene clusters. A more detailed comparison can be found in Supporting Information File 1, Table S2.

Not surprising, Hyg17 is found in an SSN cluster with known inositol dehydrogenases. To further analyze the relationship between Hyg17 and these inositol dehydrogenases, a second SSN was generated using only sequences from this cluster (Figure 4a). Enzymes with verified activities are grouped in three of the main clusters. However, Hyg17 is found in a small cluster independent of the other sequences, which could help to explain its unique substrate scope. Interestingly, this cluster is separated into two groups. All the sequences that group with Hyg17 are found in identical hygromycin A biosynthetic clusters. The second group has 12 of the 29 Hyg genes with greater than 30% amino acid identity (Figure 4b and Supporting Information File 1, Table S2). The strains that contain these biosynthetic gene clusters could be producing a hygromycin A-like compound and further investigation is required.

### Genome mining for natural product biosynthetic clusters

Since some enzymes from PF01408 are involved in natural product biosynthetic pathways, we wanted to see if sequences from this PFAM could be used to mine for potentially new natural product biosynthetic clusters. We analyzed the genomic neighborhood of these sequences and searched for domains commonly associated with natural product biosynthetic enzymes (Supporting Information File 1, Table S3). We observed that 584 sequences were near an acyl synthase domain, 340 sequences by an acyl carrier protein domain, and 1,193 sequences by a thioesterase domain. In addition, Hyg17 works together with the aminotransferase Hyg8 to replace a hydroxy group with an amine generating an aminocyclitol from *myo*-inositol. We noticed that some other enzymes from this PFAM are also paired with aminotransferases to generate amino sugars found in natural products [25–26], so we looked to see if aminotransferases are commonly found by PF01408 sequences. We observed three separate aminotransferase PFAMs near many of the PF01408 sequences. DegT/DnrJ/EryCq/StrS aminotransferases (PF01041) were most frequently associated with PF01408 sequences with 15,139 occurrences. Class III (PF00202) and class I and II (PF00155) aminotransferases were significantly less frequent, with 1,236 and 1,318 occurrences, respectively. Furthermore, the presence of a resistance gene within a biosynthetic gene cluster can indicate that it produces an antimicrobial compound [27]. We searched for annotated resistance genes in the surrounding genomic neighborhood of PF01408 sequences. We found 1,166 PF01408 sequences were associated with nearby glyoxalase/bleomycin resistance protein/dioxygenase superfamily (PF00903) sequences, 81 associated with CrcB-like protein, camphor resistance (PF02537) sequences, 22 associated with aminoglycoside antibiotic resistance kinase (PF04655) sequences, and 2 associated with putative multidrug resistance efflux protein (PF13536) sequences. Together, this suggests that PF01408 sequences may be found in a significant number of uncharacterized biosynthetic gene clusters. However, a more detailed analysis of the genomic neighborhoods is needed to assess their promising potential in natural product discovery.

## Conclusion

Hygromycin A is a broad-spectrum antibiotic that contains an aminocyclitol moiety essential for bioactivity [6–7]. The biosynthesis of the aminocyclitol has been proposed to proceed through six steps starting from glucose-6-phosphate through a *myo*-inositol intermediate to the final methylenedioxy aminocyclitol [8]. However, none of the enzymes in the pathway have been characterized in vitro. In this study, we show that Hyg17 is an NAD^+^-dependent *myo*-inositol dehydrogenase with an optimal pH of 10.5–11. When tested on a range of inositol substrate, we show that Hyg17 has reduced activity on *scyllo*-inositol and ʟ-*chiro*-inositol substrates, which is unusual for a *myo*-inositol dehydrogenase. Hyg17 belongs to PF01408, which is a protein family whose members have a broad range of activities and substrates. However, there are a variety of PF01408 members involved in various natural product biosynthetic pathways. Genome neighborhood analysis shows that many PF01408 sequences can be found near known biosynthetic enzymes, aminotransferases, and resistance genes suggesting that these enzymes may be promising candidates for targeted gene mining strategies to discover novel natural products.

## Experimental

### Cloning, expression, and purification

*Streptomyces leeuwenhoekii* NRRL B-24963 [28] was used as a template to amplify the *hyg17* (GenBank CQR59633) with the primers 5’-GTTAGCCATATGACGGTCGCCGTCGTGGGC-3’ and 5’-GTAATGCTCGAGCGGCGCCACCGGCACCGA-3’. *hyg17* was cloned into pTip-QC1 [10] using NdeI and XhoI restriction sites and verified by DNA sequencing (Eurofins Genomics). pTip-QC1-*hyg17* plasmid [10] was transformed into *Rhodococcus jostii* RHA1 [11]. Cultures were grown in Luria Bertani (LB) media supplemented with 34 µg mL^−1^ chloramphenicol at 30 °C while shaking at 200 rpm for 48 h reaching an OD_600_ of ≈1.4 then induced with 50 µL of 20 mg mL^−1^ thiostrepton and grown for another 24 h at 30 °C while shaking at 200 rpm. Cells were then harvested by centrifugation, resuspended in binding buffer (500 mM NaCl, 20 mM Tris pH8.0) and disrupted by sonication using a Branson Sonifier 450 (5 rounds of 3 s/3 s on/off cycles for 5 min at a duty cycle of 50). Initial purification was performed with gravity filtration using nickel-nitrilotriacetic acid (Ni-NTA) resin (GE Healthcare). Cell lysate was loaded onto the Ni-NTA resin and washed with binding buffer. Protein was eluted using increasing concentrations of imidazole in binding buffer (10, 20, 50, and 500 mM imidazole). Fractions were run on an ExpressPlus^TM^ PAGE Gel (GenScript) and protein was visualized using Coomassie Brilliant Blue G-250 (VWR). Fractions containing Hyg17 were pooled and further purified using a HiLoad 16/600 Superdex 75 pg size exclusion chromatography column (Cytiva) equilibrated in 50 mM NaCl, 20 mM Tris pH 8.0 and run on a Bio-Rad NGC chromatography system. Protein-containing fractions were identified by UV absorbance at 280 nm and were pooled and concentrated using a centrifugal device with a 10 kDa molecular weight cut-off (Pall Corporation).

*bsIDH* (Uniprot ID P26935) was gene synthesized with codon optimization and cloned into pET28a by Twist Bioscience. pET28a-*bsIDH* was transformed into *E. coli* BL21 star (DE3) (Agilent). For overnight cultures, 5–6 colonies were inoculated into 50 mL of LB supplemented with 10 μg mL^−1^ ampicillin and 1.5 μg mL^−1^ tetracycline and grown at 200 rpm and 37 °C overnight. One liter of autoinduction media (20 g L^−1^ tryptone, 10 g L^−1^ yeast extract, 50 mM NH_4_Cl, 2 mM MgSO_4_, 0.5% glycerol, 17 mM KH_2_PO_4_, 72 mM K_2_HPO_4_, 0.05% glucose, and 0.2% lactose) supplemented with 10 μg mL^−1^ ampicillin and 1.5 μg mL^−1^ tetracycline was inoculated with 20 mL of overnight culture and incubated at 200 rpm and 37 °C for 2 h. After 2 h, the culture was grown at 150 rpm and 16 °C for three nights. Cultures were harvested by centrifugation at 5,000 rpm for 10 min. The pellet was disrupted using a chemical lysis method. Specifically, the pellet was resuspended in 40 mL of sucrose solution (25% sucrose, 50 mM Tris pH 8) using continuous stirring. Then, 10 mg of lysozyme (Bio Basic) was added and stirred at room temperature for 10 min, followed by the addition of 80 mL deoxycholate solution (1% deoxycholate, 1% Triton X-100, 100 mM NaCl, 20 mM Tris pH 7.5) for another 10 min. Then MgSO_4_ (to ≈1 mM) and 0.2 mg DNase (Thermo Fisher Scientific) were added and stirred for 10 min. The lysate was centrifuged to remove debris for 45 min at 10,000 rpm and 4 °C, and the lysate was collected into a beaker. Purification was then performed using the same protocol as described above for Hyg17 except that a HiLoad 26/600 Superdex 200 pg size-exclusion chromatography column (Cytiva) equilibrated in 50 mM NaCl, 20 mM Tris pH 8.0 was used.

Protein concentration was determined by UV absorbance at 280 nm using the calculated extinction coefficient 33,460 M^−1^ cm^−1^ and 38,350 M^−1^ cm^−1^ for Hyg17 and BsIDH, respectively [29]. Protein was flash frozen in liquid nitrogen at 100 μM in 20 mM Tris pH 8, 50 mM NaCl, and 10% glycerol and stored at −80 °C for biochemical assays.

### Enzyme assays

Generally, 100 μL reactions contained 10 mM inositol substrate, 10 mM NAD^+^ cofactor, 1 μM Hyg17 enzyme, 100 mM CAPS, 50 mM NaCl, pH 10.5 buffer. The production of NADH was monitored by measuring the absorbance at 340 nm using the SpectraMax M2 UV–vis spectrophotometer over 20 min at 25 °C. The optimal pH was determined by performing reactions with varying buffers (Tris-HCl, HEPES, CHES and CAPS) from pH 7.5 to 11. Different inositol substrates, including *myo*-inositol, *scyllo*-inositol, *muco*-inositol, *epi*-inositol, ᴅ-*chiro*-inositol, ʟ-*chiro*-inositol, and ᴅ-glucose were tested. Inositol substrates were purchased from TCI Chemicals or Santa Cruz Biotechnology. In addition, NAD^+^ and NADP cofactors were also tested in reactions with *myo*-inositol. NAD^+^ and NADP were purchased from Research Products International. Results were analyzed using GraphPad Prism version 9.5.1 for Windows, GraphPad Software, Boston, Massachusetts USA, https://www.graphpad.com.

Reactions for thin-layer chromatography analysis were carried out using the same general reaction conditions reported above. Six μL of each reaction were spotted on the TLC silica gel 60 F_254_ (Sigma) and run with a solvent system containing butanol/ethanol/water 5:4:3. The TLC was stained using a solution containing 3.7 mL *p*-anisaldehyde, 135 mL ethanol, 5 mL sulfuric acid, and 1.5 mL glacial acetic acid. Light pink spots were observed following heating at 105 °C.

Kinetics assays were carried out by varying concentrations of substrate (*myo*-inositol or *scyllo*-inositol) from 1 mM to 60 mM with 1 μM Hyg17 and 10 mM NAD^+^ in 100 mM CAPS, 50 mM NaCl, pH 10.5. Additional kinetics assays were carried out by varying concentrations of NAD^+^ from 1 mM to 40 mM with 1 μM Hyg17 and 10 mM *myo*-inositol in 100 mM CAPS, 50 mM NaCl, pH 10.5. The production of NADH was monitored and initial reaction rates calculated by determining the slope of the reaction from 0 to 5 min. Results were analyzed using Microsoft Excel and GraphPad Prism version 9.5.1 for Windows, GraphPad Software, Boston, Massachusetts USA, https://www.graphpad.com.

### SSN network generation and genome mining

The sequence similarity network was generated using the enzyme function initiative (EFI-EST) [30] web tool. An SSN was created by inputting the Hyg17 sequence into the Sequence BLAST function and retrieving the top 500 sequence hits along with sequences from the protein family PF01408 in which Hyg17 is found. The sequences were filtered for bacterial sequences and the UniRef50 function was used along with an *E*-value cut off of 1 × 10^−55^ and node network with 80% ID. Clusters with a single node were removed for simplicity. This produced an SSN with 28,698 nodes. A second SSN was generated for the IolG cluster from the PF01408 SSN. The Uniprot IDs for all sequences found in the IolG cluster were used to generate an SSN using the EFI-EST web tool. The *E*-value cut off was set to 1 × 10^−80^ and the node network with 80% ID was used. Clusters with a single node were removed for simplicity which generated an SSN with 5339 nodes. Genomic neighborhood analysis was completed using the enzyme function initiative (EFI-GNT) web tool [31–32]. Gene clusters were compared using CAGECAT [33]. SSNs and GNNs were visualized using Cystoscape [34].

## Supporting Information

File 1 Additional tables and figures.

## Data Availability

All data that supports the findings of this study is available in the published article and/or the supporting information to this article.
