# Supplementary material for: A myo-inositol dehydrogenase involved in aminocyclitol biosynthesis of hygromycin A
Source: Beilstein J Org Chem. 2024 Mar 14;20:589–96. doi: 10.3762/bjoc.20.51 (PMC10949010; doi:10.3762/bjoc.20.51)
Supplement: File 1 — Additional tables and figures. [file Beilstein_J_Org_Chem-20-589-s001.pdf]

BEILSTEIN JOURNAL OF ORGANIC CHEMISTRY

Supporting Information

for

A *myo*-inositol dehydrogenase involved in aminocyclitol biosynthesis of hygromycin A

Michael O. Akintubosun and Melanie A. Higgins

Beilstein J. Org. Chem. **2024,** *20,* 589–596. doi:10.3762/bjoc.20.51

Additional tables and figures

License and Terms: This is a supporting information file under the terms of the Creative Commons Attribution License (https://creativecommons.org/licenses/by/4.0). Please note that the reuse, redistribution and reproduction in particular requires that the author(s) and source are credited and that individual graphics may be subject to special legal provisions.
The license is subject to the *Beilstein Journal of Organic Chemistry* terms and conditions: (https://www.beilstein-journals.org/bjoc/terms)

Table of contents

Table S1	Characterized enzymes from PF01408	S2
Table S2	Comparison of the hygromycin A biosynthetic clusters	S4
Table S3	Genomic neighborhood analysis of PF01408 sequences	S5
Figure S1	Hyg17 Purification	S6
Figure S2	pH activity profile	S7
Figure S3	TLC analysis of Hyg17 reaction with *myo*-inositol	S8

Table S1: Characterized enzymes from PF01408.

Gene name	Uniprot ID	Function	Biological role	Reference
**Inositol metabolism**				
IolG	P26935	*Myo*-inositol 2-dehydrogenase	*Myo*-inositol catabolism	[1]
	Q9WYP5	*Myo*-inositol 2-dehydrogenase	*Myo*-inositol catabolism	[2]
	O68965	*Myo*-inositol 2-dehydrogenase	*Myo*-inositol catabolism	[3]
	Q8NTY7	*Myo*-inositol 2-dehydrogenase	*Myo*-inositol catabolism	[4]
	P40332	*Scyllo*-inositol 2-dehydrogenase	*Scyllo*-inositol catabolism	[5]
	Q8NNS7	*Scyllo*-inositol 2-dehydrogenase	*Scyllo*-inositol catabolism	[4]
IolU	O05265	*Scyllo*-inositol 2-dehydrogenase	*Scyllo*-inositol catabolism	[6]
IolW	O32223	*Scyllo*-inositol 2-dehydrogenase	*Scyllo*-inositol catabolism	[6]
	Q8NTY4	*Scyllo*-inositol 2-dehydrogenase	*Scyllo*-inositol catabolism	[4]
**Natural product biosynthesis**				
KijD10	B3TMR8	dTDP-3,4-didehydro-2,6-dideoxy-alpha-D-glucose 3-reductase	L-Digitoxose biosynthesis	[7]
SpnN	Q9ALN5	dTDP-3,4-didehydro-2,6-dideoxy-alpha-D-glucose 3-reductase	Forosamine biosynthesis	[8]
OleW	Q9RR32	dTDP-3,4-didehydro-2,6-dideoxy-alpha-D-glucose 3-reductase	L-Oleandrose biosynthesis	[9]
IgdH	F0M433	Levoglucosan dehydrogenase	Levoglucosan biosynthesis	[10]
NtdC	O07564	Glucose-6-phosphate 3-dehydrogenase	Kanosamine biosynthesis	[11]
KanD2	Q6L737	Glucose-6-phosphate 3-dehydrogenase	Kanosamine biosynthesis	[12]
RifL	Q7BUE1	UDP-glucose dehydrogenase	Kanosamine biosynthesis	[13]
Irp3	W9BA38	Thiazolinyl imine reductase	Yersiniabactin biosynthesis	[14]
**Carbohydrate metabolism**				
NagA	A4Q8F7	α-N-Acetylgalactosaminidase	Blood group antigen degradation	[1-5][15]
	A4Q8G1	α-N-Acetylgalactosaminidase	Blood group antigen degradation	[15]
	Q8ECL7	α-N-Acetylgalactosaminidase	Blood group antigen degradation	[15]
AraA	Q53TZ2	L-arabinose 1-dehydrogenase	L-Arabinose degradation	[16]
Gal	P11886	Galactose 1-dehydrogenase	Galactose metabolism	[17]

ApsD	B1G894	Apiose dehydrogenase	D-Apiose catabolism	[18]
	B9JK80	Apiose dehydrogenase	D-Apiose catabolism	[18]
Xdh	Q8GAK6	D-Xylose 1-dehydrogenase	D-Xylose degradation	[19]
	Q5UY95	D-Xylose 1-dehydrogenase	D-Xylose catabolism	[24–26][20]
PLH35	T2KNC8	Oxidoreductase P35	Ulvan degradation	[21]
Gfo	A0A143PP41	Aldose-Aldose Oxidoreductase	Unknown	[22]
YjhC	P39353	2,7-anhydro-Neu5Ac dehydrogenase	Sialic acid metabolism	[23]
YcjS	P77503	Glucoside 3-dehydrogenase	Unknown carbohydrate catabolism	[24]
**LPS biosynthesis**				
WbpB	G3XD23	UDP-N-acetyl-2-amino-2-deoxy-D-glucuronate dehydrogenase	LPS biosynthesis.	[25–27]
WlbA	I6UWH1	UDP-N-acetyl-2-amino-2-deoxy-D-glucuronate dehydrogenase	LPS biosynthesis	[28]
GnnA	A0A543Q2K9	UDP-N-acetylglucosamine 3-dehydrogenase	LPS biosynthesis	[29]
**Other functions**				
PhtC	Q05184	Phthalate cis-4,5-dihydrodiol dehydrogenase	Cis-3,4-dihydrodiol phthalate catabolism	[30]
DgpA	A0A3Q9WWX8	C and O glycoside isomerase	Puerarin catabolism	[31]
LigC	Q9KWL3	4-carboxy-2-hydroxymuconate-6-semialdehyde dehydrogenase	Protocatechuate catabolism	[32]
Ddh	Q5L9Q6	*m*-Diaminopimelate dehydrogenase	L-Lysine biosynthesis	[33]

Table S2: Comparison of the hygromycin A biosynthetic cluster [34] and unknown hygromycin A-like cluster.

Protein	Annotated function	HygA biosynthetic role	% ID to unknown cluster
Hyg1	AfsR regulatory protein	Regulation	---
Hyg2	*p*-Hydroxybenzoate hydroxylase	Cinnamic acid	---
Hyg3	Regulatory protein	Regulation	---
Hyg4	Chorismate lyase	Cinnamic acid	---
Hyg5	Mannose dehydratase	Furanose	---
Hyg6	Methyltransferase	Aminocyclitol	56
Hyg7	Unknown	Aminocyclitol	69
Hyg8	Aminotransferase	Aminocyclitol	65
Hyg9	ACP	Cinnamic acid	31
Hyg10	b-ketoacyl synthase I	Cinnamic acid	50
Hyg11	Unknown	Unknown	---
Hyg12	CoA-ligase	Cinnamic acid	37
Hyg13	ACP	Cinnamic acid	
Hyg14	3-Hydroxyacyl ACP dehydratase	Cinnamic acid	34
Hyg15	3-Hydroxyacyl ACP reductase	Cinnamic acid	44
Hyg16	Glycosyltransferase	Furanose attachment	45
Hyg17	*Myo*-inositol dehydrogenase	Aminocyclitol	50
Hyg18	*Myo*-inositol-1-phosphate synthase	Aminocyclitol	---
Hyg19	Transmembrane protein	Unknown	---
Hyg20	Transglucosylase	Furanose	57
Hyg21	Phosphotransferase	Resistance	37
Hyg22	Acyltransferase	Cinnamic acid	---
Hyg23	Fucose synthase	Furanose	32
Hyg24	Unknown	Unknown	
Hyg25	*Myo*-inositol-1-phosphatase	Aminocyclitol	---
Hyg26	Short chain dehydrogenase	Furanose	---
Hyg27	DAHPS synthase	Cinnamic acid	---
Hyg28	ABC transporter	Unknown	---
Hyg29	Methyltransferase	Unknown	---

Table S3: Genomic neighborhood analysis of PF01408 sequences.

PFAM	Function	# Sequences with PFAM neighbors	# SSN clusters that contain PFAM
	**NP biosynthetic enzymes**		
PF00109	Ketoacyl synthase	584	45
PF00550	Acyl carrier protein (ACP) domain	340	71
PF00975	Thioesterase (TE) domain*	1,193	22
	**Aminotransferases**		
PF01041	DegT/DnrJ/EryCq/StrS aminotransferase	15,139	340
PF00202	Aminotransferase – class III	1,236	149
PF00155	Aminotransferase – class I and II	1,318	136
	**Putative resistance proteins**		
PF04655	Aminoglycoside antibiotic resistance kinase	22	4
PF02537	CrcB-like protein, Camphor Resistance	81	5
PF00903/ PF13660	Glyoxalase/Bleomycin resistance protein/Dioxygenase superfamily	1354	160
PF13536	Putative multidrug resistance efflux protein	2	2
PF05099	Tellurite resistance protein TerB	8	5

*TE domains only on their own. Some other TE domains are also found with ACP domains.

Figure S1: SDS-PAGE gel of purified Hyg17.

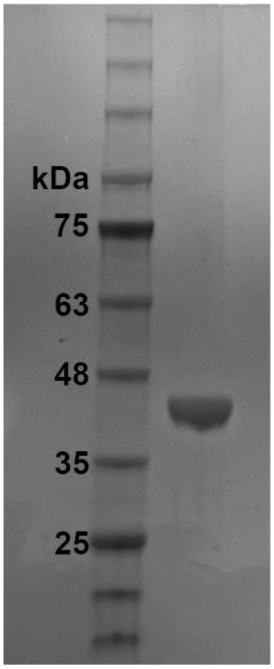

Expected MW
36 kDa

Figure S2: pH Activity profile for reactions with 10 mM *myo*-inositol, 10 mM NAD^+, 1 μM Hyg17, 100 mM HEPEs, 100 mM Tris, 100 mM CHES, and 100 mM CAPS, 50 mM NaCl, pH 9.5–11.

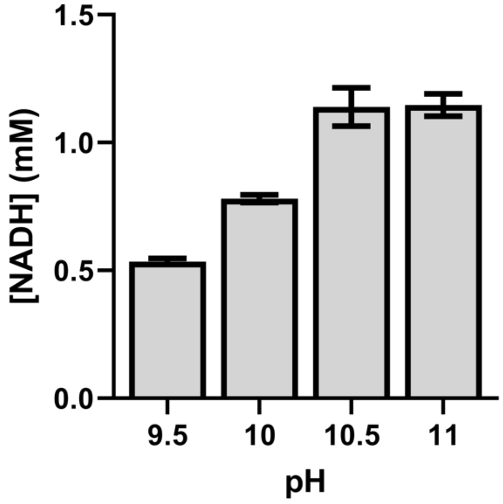

Figure S3: TLC analysis for Hyg17 and BsIDH reactions with *myo*-inositol and NAD^+. *p*-Anisaldehyde was used to stain ketones light pink.

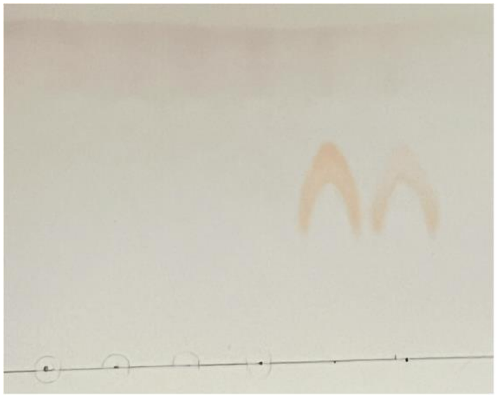

-	+	-	+	+	+	NAD^+
-	-	+	+	+	+	*myo*-inositol
-	-	-	-	+	-	Hyg17
-	-	-	-	-	+	BsIDH

References:

1. Ramaley, R.; Fujita, Y.; Freese, E. *J Biol Chem* ,**1979**, *254*, 7684–7690
2. Rodionova, I. A.; Leyn, S. A.; Burkart, M. D.; Boucher, N.; Noll, K. M.; Osterman, A. L.; Rodionov, D. A. *Environ Microbiol* **2013**, *15*, 2254–2266.
3. Galbraith, M. P.; Feng, S. F.; Borneman, J.; Triplett, E. W.; De Bruijn, F. J.; Rossbachl, S. *Microbiology,* **1998**, *144*, 2915–2924.
4. Ramp, P.; Pfleger, C.; Dittrich, J.; Mack, C.; Gohlke, H.; Bott, M. *Microbiol Spectr* **2022**, *10*, e01950-22.
5. Morinaga, T.; Ashida, H.; Yoshida, K. *Microbiology,* **2010**, *156*, 1538–1546.
6. Kang, D.-M.; Tanaka, K.; Takenaka, S.; Ishikawa, S.; Yoshida, K. *Bioscience, Biotechnology, and Biochemistry,* **2017**, *81*, 1026–1032.
7. Kubiak, R. L.; Holden, H. M. *Biochemistry,* **2011**, *50*, 5905–5917.
8. Waldron, C.; Matsushima, P.; Rosteck, P. R.; Broughton, M. C.; Turner, J.; Madduri, K.; Crawford, K. P.; Merlo, D. J.; Baltz, R. H. *Chemistry & Biology,* **2001**, *8*, 487–499.
9. Aguirrezabalaga, I.; Olano, C.; Allende, N.; Rodriguez, L.; Braña, A. F.; Méndez, C.; Salas, J. A. *Antimicrob Agents Chemother,* **2000**, *44*, 1266–1275.
10. Sugiura, M.; Nakahara, M.; Yamada, C.; Arakawa, T.; Kitaoka, M.; Fushinobu, S. *Journal of Biological Chemistry*, **2018**, *293*, 17375–17386.
11. Vetter, N. D.; Langill, D. M.; Anjum, S.; Boisvert-Martel, J.; Jagdhane, R. C.; Omene, E.; Zheng, H.; Van Straaten, K. E.; Asiamah, I.; Krol, E. S.; Sanders, D. A. R.; Palmer, D. R. J. *J. Am. Chem. Soc.,* **2013**, *135*, 5970–5973.
12. Kudo, F.; Kitayama, Y.; Miyanaga, A.; Hirayama, A.; Eguchi, T. *Biochemistry,* **2020**, *59*, 1470–1473.
13. Yu, T.-W.; Müller, R.; Müller, M.; Zhang, X.; Draeger, G.; Kim, C.-G.; Leistner, E.; Floss, H. G. *Journal of Biological Chemistry,* **2001**, *276*, 12546–12555.
14. Meneely, K. M.; Lamb, A. L. *Biochemistry,* **2012**, *51*, 9002–9013.
15. Liu, Q. P.; Sulzenbacher, G.; Yuan, H.; Bennett, E. P.; Pietz, G.; Saunders, K.; Spence, J.; Nudelman, E.; Levery, S. B.; White, T.; Neveu, J. M.; Lane, W. S.; Bourne, Y.; Olsson, M. L.; Henrissat, B.; Clausen, H. *Nat Biotechnol,* **2007**, *25*, 454–464.
16. Watanabe, S.; Kodak, T.; Makino, K. *Journal of Biological Chemistry,* **2006**, *281*, 2612–2623.
17. Blachnitzky, E.; Wengenmayer, F.; Kurz, G. *European Journal of Biochemistry,* **1974**, *47*, 235–250.
18. Carter, M. S.; Zhang, X.; Huang, H.; Bouvier, J. T.; Francisco, B. S.; Vetting, M. W.; Al-Obaidi, N.; Bonanno, J. B.; Ghosh, A.; Zallot, R. G.; Andersen, H. M.; Almo, S. C.; Gerlt, J. A. *Nat Chem Biol,* **2018**, *14*, 696–705.
19. Mihasan, M.; Stefan, M.; Hritcu, L.; Artenie, V.; Brandsch, R. *Research in Microbiology,* **2013**, *164*, 22–30.
20. Johnsen, U.; Schönheit, P. *J Bacteriol,* **2004**, *186*, 6198–6207.
21. Bäumgen, M.; Dutschei, T.; Bartosik, D.; Suster, C.; Reisky, L.; Gerlach, N.; Stanetty, C.; Mihovilovic, M. D.; Schweder, T.; Hehemann, J.-H.; Bornscheuer, U. T. *Journal of Biological Chemistry*, **2021**, 101210.

22. Taberman, H.; Andberg, M.; Koivula, A.; Hakulinen, N.; Penttilä, M.; Rouvinen, J.; Parkkinen, T. *Biochemical Journal*, **2015**, *472*, 297–307.
23. Horne, C. R.; Kind, L.; Davies, J. S.; Dobson, R. C. J. *Proteins* **2020**, *88*, 654–668.
24. Mukherjee, K.; Huddleston, J. P.; Narindoshvili, T.; Nemmara, V. V.; Raushel, F. M. *Biochemistry,* **2019**, *58*, 1388–1399.
25. Westman, E. L.; Preston, A.; Field, R. A.; Lam, J. S. *J Bacteriol,* **2008**, *190*, 6060–6069.
26. Westman, E. L.; McNally, D. J.; Charchoglyan, A.; Brewer, D.; Field, R. A.; Lam, J. S. *Journal of Biological Chemistry,* **2009**, *284*, 11854–11862.
27. Larkin, A.; Imperiali, B. *Biochemistry,* **2009**, *48*, 5446–5455. doi:10.1021/bi900186u
28. Thoden, J. B.; Holden, H. M. *Biochemistry,* **2010**, *49*, 7939–7948.
29. Manissorn, J.; Sitthiyotha, T.; Montalban, J. R. E.; Chunsrivirot, S.; Thongnuek, P.; Wangkanont, K. *ACS Chem. Biol.,* **2020**, *15*, 3235–3243.
30. Mahto, J. K.; Sharma, M.; Neetu, N.; Kayastha, A.; Aggarwal, S.; Kumar, P. *Archives of Biochemistry and Biophysics*, **2022**, *727*, 109314.
31. He, P.; Wang, S.; Li, S.; Liu, S.; Zhou, S.; Wang, J.; Tao, J.; Wang, D.; Wang, R.; Ma, W. *Acta Pharmaceutica Sinica B,* **2023**, *13*, 246–255.
32. Masai, E.; Momose, K.; Hara, H.; Nishikawa, S.; Katayama, Y.; Fukuda, M. *J Bacteriol,* **2000**, *182*, 6651–6658.
33. Hudson, A. O.; Klartag, A.; Gilvarg, C.; Dobson, R. C. J.; Marques, F. G.; Leustek, T. *Biochimica et Biophysica Acta (BBA) - Proteins and Proteomics,* **2011**, *1814*, 1162–1168.
34. Palaniappan, N.; Ayers, S.; Gupta, S.; Habib, E.-S.; Reynolds, K. A. *Chemistry & Biology,* **2006**, *13*, 753–764.
